# Wonderful Restoration of an Afflicted Minister

**Published:** 1873-05

**Authors:** Hiram Stears

**Affiliations:** Vandalia, Ill.


### WONDERFUL RESTORATION OF AN AFFLICTED MINISTER.

The Rev. J. D. Crum, a member of the Southern Illinois Conference of the Methodist Episcopal Church, now laboring on the Pleasant Grove Circuit, in the bounds of the Vandalia District, had been afflicted with something like sore throat for ten years before he had to give up the active work of the ministry some four years ago. He settled on a little farm five or six miles from this city. His health continued to decline for two years, and his neighbors all said he must die.

As a last effort to regain his health, he left his home for Cincinnati to consult, the most experienced physicians there. Several were consulted but to no purpose. He grew worse for three weeks, when his brother was summoned from Circleville, Ohio, to remove him if possible, or to see him die. He succeeded in transferring him to his home in this city, and Dr. W. Griswold was called to visit him. The disease up to this time had deceived many of the best practitioners in the country.

Some thought it was the result of Diphtheria, others that it was a singular and protracted case of Tonsilitis, while others again thought it an aggravated case of Catarrh. The patient complained of a sharp cutting sensation above the palate, as well as general inflammation of all the adjacent parts. Dr. Griswold conceived that Necrosis of the Ethmoid bone might be followed by an exfoliation which would produce in the flesh, the sharp pains complained of.

He therefore made an examination with an instrument and found that the process of exfoliation of the diseased bone or bones in that region was actually going on. One or two pieces of bone making their way through the flesh could be distinctly felt. The Doctor applied diluted Carbolic acid to the affected parts and gave the patient tonics to brace up his system. In eleven weeks Mr. Crum was fully restored to health, but with a loss of several pieces of bone and of his voice. Among the bones were also the palatal which from their proximity to the affected parts above, and contact with the matter from their protracted suppuration, had become similarly affected. In coming away, these bones had cut two clefts through the fleshy parts of the roof of the palate. The one in front is about three quarters of an inch wide. The other in the rear is perhaps three eighths of an inch in diameter.

These apertures through the roof of the mouth so destroyed the voice, that Mr. Crum had often in conversation to resort to pencil and paper to make himself understood. In this deplorable situation he suggested to us his intention to apply to a dentist at a distance, with whom he was favorably acquainted to secure a set of teeth, which he needed, with a plate running sufficiently back to cover clefts, he hoping at least for a partial restoration of his voice.

We begged the privilege of introducing him to Dr. W. B. Pike, of this city, feeling satisfied from the Doctor's reading and mechanical genius that he would succeed in this matter if any dentist could. When we applied to him, he answered very discouragingly. But he examined the patient at his earliest opportunity, and went to work. How to get up the proper amount of suction with these openings in the roof of the mouth was the difficulty. To overcome this seemingly insuperable difficulty, a good sized and well fitted plate was inserted, sufficiently large enough to cover both openings completely, but not to interfere too much with the flexible portion of the palate which still remained. The gums of the upper teeth were well corrugated on the outside to increase the suction in that direction. The teeth were adjusted with great care and precision. And to the agreeable surprise of Dr. Pike himself, and of all the medical faculty in the city, his experiment was successful. The moment the teeth and plate were inserted in the mouth the patient's voice was completely restored. Mr. Crum converses and preaches with as much ease and with as clear and distinct a voice as he ever did. He has resumed the active work of the ministry again, full of zeal, and no less abounding in gratitude to Dr. Pike, who by the singular triumph in the practice of dentistry has won a place and a name among his compeers second to none in this country. HIRAM STEARS.

Vandalia, Ill., Oct. 9th, 1871.

